# Pharmacological Effect of Water-Extractable (Poly)Phenolic Polysaccharide–Protein Complexes from *Prunus spinosa* L. Wild Fruits

**DOI:** 10.3390/ijms26135993

**Published:** 2025-06-22

**Authors:** Šutovská Martina, Miroslava Molitorisová, Jozef Mažerik, Iveta Uhliariková, Peter Capek

**Affiliations:** 1 Department of Pharmacology, Jessenius School of Medicine in Martin, Comenius University in Bratislava, Malá Hora 11161/4B, SK-03601 Martin, Slovakia; martina.sutovska@uniba.sk (Š.M.); miroslava.molitorisova@uniba.sk (M.M.); mazerik1@uniba.sk (J.M.); 2 Institute of Chemistry, Slovak Academy of Sciences, Dúbravská cesta 9, SK-845 38 Bratislava, Slovakia; chemivuh@savba.sk

**Keywords:** *Prunus spinosa*, polysaccharide complex, cough reflex, bronchodilatory effect

## Abstract

Wild fruits are distributed worldwide, but are consumed mainly in developing countries, where they are an important part of the diet. Still, in many other countries, they are consumed only locally. Blackthorn (*Prunus spinosa* L.) is an underutilized species rich in fibres and phenolic compounds, making it suitable as a potential functional food for supporting human health. Cold (Cw) and hot (Hw) water-extracted (poly)phenolic polysaccharide–protein complexes, differing in carbohydrate, phenolic and protein contents, were isolated from blackthorn fruits and characterized. The complexes exhibited molecular weights of 235,200 g/mol (Cw) and 218,400 g/mol (Hw), and were rich in pectic polymers containing galacturonic acid, arabinose, galactose and rhamnose, indicating a dominance of homogalacturonan (HG) [→4)-α-D-GalA(1→4)-α-D-GalA(1→]n and a low content of RGI [→2)-α-L-Rha(1→4)-α-D-GalA(1→2)-α-L-Rha(1→]n sequences associated with arabinan or arabinogalactan. Minor content of glucan, probably starch-derived, was also solubilized. Pectic polysaccharides were highly esterified and partly acetylated. Pharmacological testing was performed in male Dunkin–Hartley guinea pigs, a model with human-like airway reflexes. Both complexes affected airway defense mechanisms. Particularly, Hw significantly suppressed citric acid-induced cough, similar to codeine, and reduced bronchoconstriction comparably to salbutamol in a dose-dependent manner. These findings support further exploration of Hw as a natural antitussive and bronchodilatory agent.

## 1. Introduction

The blackthorn (*Prunus spinosa* L.) is a spiny deciduous shrub that produces small purple edible plums. This species is mainly found from southern central Europe to southern Scandinavia and east to Asia Minor, growing on forest edges and open woodlands as part of Mediterranean thermophilic plant communities. It is mostly wild-growing or cultivated as an ornamental plant for its fruits and is used to make jams, wine, vinegar and spirits [1]. *P. spinosa* has several notable medicinal applications due to its rich composition of bioactive compounds found in its flowers, fruits and leaves. The flowers are recommended as a complementary treatment for diseases related to oxidative stress, and they exhibit antioxidant activity against the primary in vivo relevant reactive oxygen species. Their extracts are a rich source of polyphenols, especially flavonoids and phenolic acids [2,3], which are considered bioactive substances with potent anticancer effects [4].

Similarly, their fruits are a rich source of flavonoids, anthocyanins, phenolic acids, glycoconjugates, vitamins, minerals and organic acids, which exhibit significant antioxidant and antibacterial properties [5]. Flavonoids such as catechin, epicatechin and rutin have been reported to have protective effects against diabetes, while other flavonoids, including myricetin, quercetin and kaempferol, exhibit antihypertensive activity [6].

Extracts from leaves have antioxidant properties and show antibacterial effects, especially against *Bacillus cereus* and *Escherichia cloacae* [7]. The extracts also show inhibitory activity against enzymes associated with type II diabetes mellitus [8] and antitumor effects on malignant cell lines [9]. The main compounds identified in the *P. spinosa* leaf and flower extract mixture include chlorogenic acid and ellagic acid, contributing to its antioxidant capacity [7,9].

Further, *P. spinosa* has been used in traditional medicine for various purposes, including the treatment of gastric ulcers. Ethanol extracts of fruits have shown antioxidant, anti-inflammatory and wound-healing properties in a rat model of indomethacin-induced gastric ulcers [10]. Extracts of flowers are traditionally indicated for the treatment of urinary tract disorders, inflammation and cardiovascular diseases. They have been found to have antioxidant activity, potential anti-inflammatory effects, and cellular safety [11].

Given the existing data in the literature on anti-inflammatory effects associated with various components of *P. spinosa* fruits, we focused on investigating the influence of phenolic polysaccharide–protein complexes on airway defense reflexes, such as cough and bronchoconstriction. We hypothesized that the fruit extracts, rich in bioactive compounds, would suppress citric acid-induced cough and reduce bronchoconstriction. These reflexes are commonly associated with oxidative stress and inflammation, suggesting a possible interplay with the antioxidant properties of *P. spinosa* fruit. Guinea pigs were selected for the in vivo experiments due to their well-characterized cough reflex and airway responsiveness, which closely resemble those of humans, making them a relevant model for translational respiratory research.

## 2. Results and Discussion

### 2.1. Plant Material and Characterization of (Poly)Phenolic Polysaccharide–Protein Complexes

(Poly)phenolic polysaccharide–protein complexes were isolated from the cell walls of ripe *P. spinosa* L. fruits by sequential extraction with cold (Cw) and hot (Hw) water in a yield of 1.0 wt% and 3.8 wt%, respectively (Table 1). Both water fractions showed relatively broad molecular weight distribution patterns but differed in the contents of carbohydrates, protein, phenolics and uronic acid. The Hw fraction was richer in proteins and phenolics and had a significantly higher yield. Both blackthorn fractions were rich in uronic acids (~34 wt% (Cw) and 39 wt% (Hw)), while the content of rhamnose residues in fractions was only about 4 wt%. This fact indicates the dominance of pectic polysaccharide–homogalacturonan in these water-extracted fractions. In addition, the second dominant polymers were arabinan or arabinogalactan, which represented 17 wt% of the Cw fraction and about 20 wt% of the Hw fraction. Low levels of glucans, probably derived from starch, were also solubilized in both fractions [5].

### 2.2. NMR of the (Poly)Phenolic Polysaccharide–Protein Complex Hw

**Figure 1:**
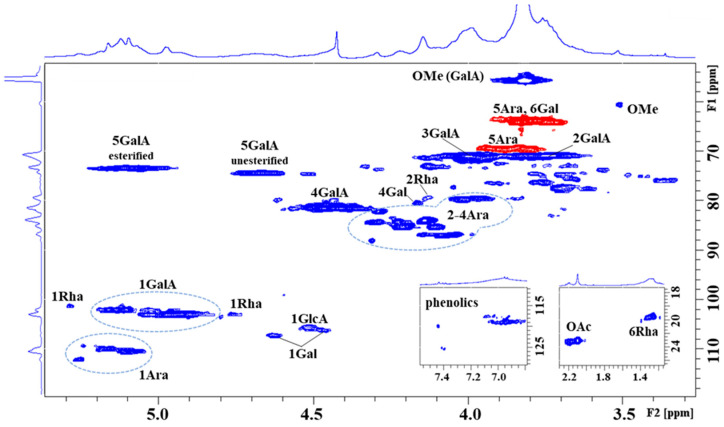
Selected regions of ^1^H-^13^C hetero-correlated HSQC spectra (CH—blue colour; CH_2_—red colour) of the hot water fraction (Hw) of blackthorn fruits.

The HSQC spectra of both fractions were similar, indicating identical pectin material extracted with cold and hot water. Differences were observed mainly in the yield of the fractions, with the hot water yield being four times higher than that with cold water [5]. The HSQC spectrum (selected regions—Figure 1, and full spectrum—Figure S2 in the Supplementary Data) of the blackthorn hot water extract (Hw) showed characteristic signals of homogalacturonan (HG), rhamnogalacturonan (RG-I), and arabinogalactan (AG), which are characteristic components of pectin [12,13]. In the α-anomeric region, the most intense broad H1/C1 signals at δ_H1/C1_ 5.135/102.09, 5.039/102.97, 4.969/102.98 ppm were assigned to 1,4-linked α-D-Gal*p*A residues and its methyl ester present in different environments of galacturonan. The characteristic H5/C5 signal at δ_H5/C5_ 5.090/73.38 ppm and the methyl group signal at δ_H/C_ 3.819/55.67 ppm confirmed the esterification of α-D-Gal*p*A residues. The other H5/C5 signal at δ_H5/C5_ 4.676/74.35 ppm originated from unesterified α-D-Gal*p*A residues. The ratio of esterified to unesterified residues 72:28 indicated a higher degree of esterification. The observed H4/C4 signals at δ_H4/C4_ 4.464/81.54, 4.408/81.52 ppm derive from 1,4-linked esterified and unesterified α-D-Gal*p*A, respectively. The remaining skeletal H2/C2 and H3/C3 signals of both esterified and unesterified α-D-GalpA were found at δ_H2/C2_ 3.740/70.86, and δ_H3/C3_ 3.998/71.0 ppm. In addition, the less intense α-anomeric signal at δ_H1/C1_ 5.286/101.20 ppm and skeletal signals at δ_H2/C2_ 4.131/79.38 and δ_H6/C6_ 1.257/19.48 ppm were attributed to 1,2-linked α-L-Rha*p* residue, as constituents of rhamnogalacturonan. The presence of acetylated sugar residue is a feature of pectin. In the region of ~ δ_H_ 2.227–2.096 ppm, four CH_3_ signals of the acetyl group were detected, with the dominant one at δ_H/C_ 2.096/23.04 ppm, thereby revealing the presence of acetylated α-D-Gal*p*A residue at position O3 [14]. Further, in the α-anomeric region, a group of signals (~ δ_C1_ 109–112 ppm), belonging to α-L-Ara*f* residues were observed. We were able to identify 1,5-linked (δ_H1/C1_ 5.090/110.40 ppm), 1,3,5-linked (δ_H1/C1_ 5.115/110.38 ppm), and four terminally linked (δ_H1/C1_ 5.158/109.97, 5.184/109.91, 5.244/109.27, 5.257/112.0 ppm) α-L-Ara*f* residues, suggesting the presence of highly branched arabinan/arabinogalactan, as a side chain of galacturonan. In the skeletal region, characteristic C2-C4 signals of Ara residues in furano-forms were present at ~ δ_C2–C4_ 79.5–88 ppm. The 1,5-linkage of α-L-Ara*f* residue was confirmed by its downfield-shifted C5 atom (δ_C5_ 69.59 ppm) with respect to the terminally linked Ara*f* residues (δ_C5_ 64.05 ppm). Finally, in the β-anomeric region, signals at δ_H1/C1_ 4.630/107.14, δ_H1/C1_ 4.465/106.18, and δ_H1/C1_ 4.512/105.53 ppm were attributed to 1,4-linked and terminally linked β-D-Gal*p* and 1,4-linked β-D-Glc*p*A residues, respectively, which together with the terminally linked α-L-Rha*p* residue (δ_H1/C1_ 4.753/103.01 ppm) are components of arabinogalactan.

### 2.3. The Evaluation of Cw and Hw Impact on the Defence Reflexes of the Airways

#### 2.3.1. The Antitussive Effect of Cw and Hw Complexes

The pharmacodynamic aspect of this study focused on evaluating the effects of *P. spinosa*-originated cold water-extracted (Cw) and hot water-extracted (Hw) glycoconjugates and codeine on the cough reflex, which was experimentally induced using CA aerosol inhalation. A guinea pig model was employed due to its well-defined cough reflex, which closely resembles that of humans, making it a widely used model in respiratory research [15]. The mechanism behind acid-induced coughing is largely attributed to the activation of vagal afferent C fibers containing tachykinins, which innervate the airways [16].

**Figure 2:**
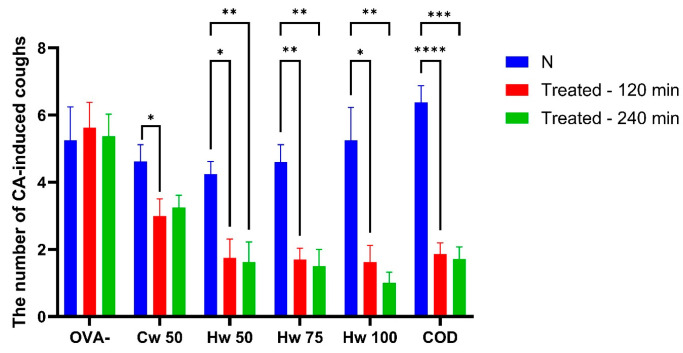
The effect of orally administered *P. spinosa* complexes Cw (50 mg/kg bw) and Hw (50, 75 and 100 mg/kg bw), codeine phosphate (10 mg/kg bw, COD), and saline (1 mL/kg bw; OVA-) on the number of citric acid (CA)-induced cough efforts in guinea pigs was evaluated. The baseline measurement (N) was recorded before drug administration. Statistical significance was determined using a one-way ANOVA followed by the Bonferroni post hoc test, with * *p* < 0.05, ** *p* < 0.01, *** *p* < 0.001 and **** *p* < 0.0001 compared to baseline (N).

Cw and Hw were administered orally, Cw at the screening dose of 50 mg/kg and Hw at doses of 50, 75 and 100 mg/kg. Their antitussive potential was compared with codeine, a centrally acting opioid cough suppressant. The findings revealed a significant reduction in the number of cough efforts following both complexes’ administrations, with a more significant impact caused by the Hw (exhibiting a dose-dependent effect). As illustrated in Figure 2, all tested doses of Hw markedly suppressed cough efforts at 60 min (*p* < 0.05 or *p* < 0.01, respectively), with this significant effect persisting throughout the experiment (*p* < 0.01). Similarly, the reference drug codeine effectively reduced cough frequency during the CA aerosol challenge. Notably, as observed in Figure 2, the cough-suppressing properties of Hw were comparable to those of the reference opioid antitussive, codeine.

#### 2.3.2. The Bronchodilatory Effect of Cw and Hw Complexes

**Figure 3:**
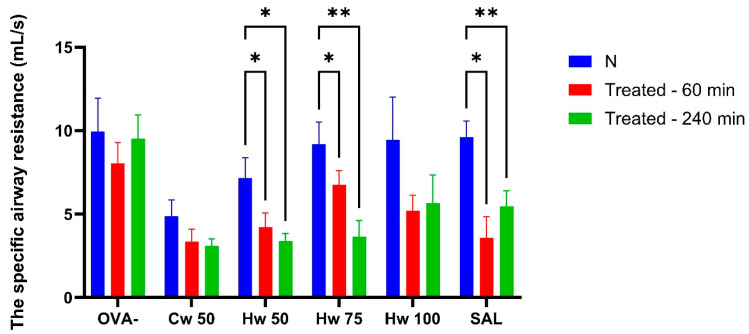
Alterations in specific airway resistance (sRaw) adjusted for respiratory rate (RR) following AC exposure assessed at baseline and at 60 and 240 min post-administration of a cold water complex (Cw) of *P.spinosa* at a dose 50 mg/kg (Cw 50) and a hot water complex (Hw) at three different doses (Hw 50, Hw 75, Hw 100), salbutamol (SAL), and saline (OVA-). Statistical analysis was performed using a one-way ANOVA with Bonferroni’s post hoc test, with * *p* < 0.05 and ** *p* < 0.01 considered significant compared to baseline (N). The baseline measurement (N) was recorded before drug administration.

**Figure 4:**
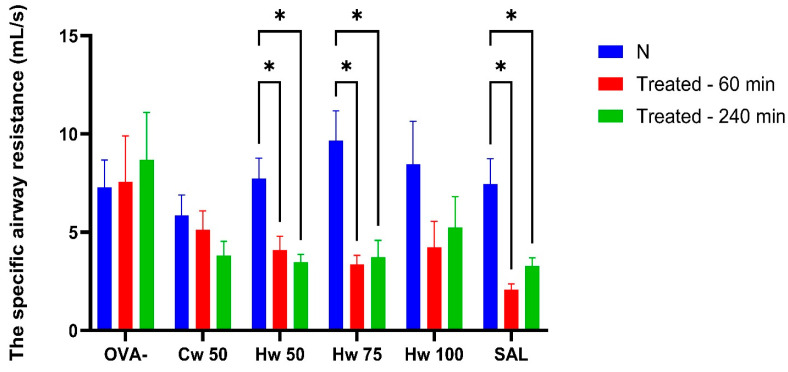
The effect of Cw and Hw complexes and control drugs on sRaw values induced by histamine. Statistical significance was determined using a one-way ANOVA followed by the Bonferroni post hoc test, with * *p* < 0.05 compared to baseline (N).

**Figure 5:**
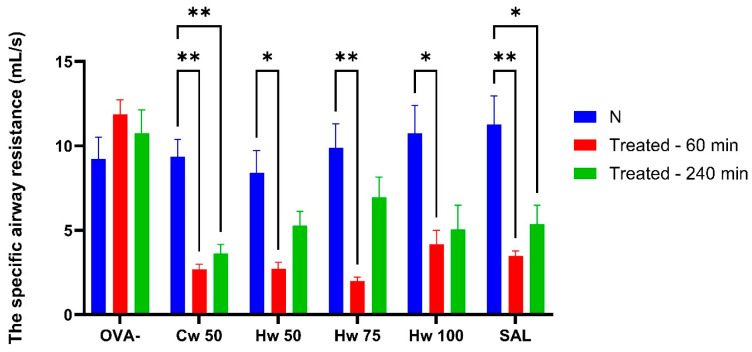
The effect of glycoconjugates and control drugs on sRaw values induced by methacholine. Statistical significance was determined using a one-way ANOVA followed by the Bonferroni post hoc test, with * *p* < 0.05 and ** *p* < 0.01 compared to baseline (N).

Plethysmographic assessment of specific airway resistance (sRaw) is a well-established and validated technique for evaluating airway obstruction, requiring no active participation from the subject. As a result, it is considered a highly sensitive and appropriate method for fundamental research and clinical monitoring [17]. In this study, sRaw measurements revealed the ability of *P. spinosa* complexes Cw and Hw to significantly inhibit airway smooth muscle contraction induced by the mediator of allergy—histamine, methacholine—thus mimicking the effect of parasympathetic acetylcholine and CA, which triggered bronchoconstriction through the release of tachykinins (Figure 3, Figure 4 and Figure 5). These mechanisms play a role not only in normal airway smooth muscle responsiveness but also in airway hyperreactivity. For comparative analysis, the commonly used bronchodilator salbutamol, a beta-2 adrenergic receptor agonist, was tested under identical experimental conditions.

Figure 3 illustrates that Hw significantly and dose-dependently lowered sRaw values that had been elevated due to short-term airway irritation caused by CA, and this effect was similar to that of the standard bronchodilator, salbutamol. The role of acidic mediators in the development of both acute and chronic airway inflammation is generally accepted [18], and reducing their impact on airway responsiveness is beneficial in conditions such as asthma and other allergic diseases. These results pointed to the potential benefit of the *P. spinosa* Hw complex.

The different effects of Cw and Hw on airway smooth muscle reactivity elicited by histamine are demonstrated in Figure 4. Cw showed no significant impact on histamine-induced bronchoconstriction, suggesting a lack of interaction with histaminergic pathways. In contrast, Hw exerted a pronounced bronchodilatory effect at doses of 50 mg/kg and 75 mg/kg body weight, with efficacy comparable to the control drug salbutamol. Since Hw is structurally distinct from salbutamol, it likely acts through mechanisms independent of beta-2 receptor activation, potentially offering therapeutic value in patients with allergic asthma refractory to beta-2 agonists as an adjunctive treatment, especially for those seeking natural therapeutic alternatives.

Methacholine mimics the effect of the natural parasympathetic mediator acetylcholine. However, methacholine is preferred over acetylcholine in airway research because it is more stable, selectively stimulates M3 muscarinic receptors, and produces a reproducible bronchoconstrictive response suitable for diagnostic and experimental use [19]. Unlike bronchoconstriction elicited by CA or histamine, the Cw complex demonstrated a significant inhibitory effect on methacholine-induced airway reactivity that lasted 240 min after its peroral administration. Additionally, the observation that the Hw significantly reduces methacholine-induced airway hyperresponsiveness (Figure 5) at all doses tested, achieving a level of bronchoprotection comparable to that of salbutamol, confirms a clinically relevant bronchodilatory effect of *P. spinosa* complexes and supports their potential therapeutic application in obstructive airway diseases [20].

Despite these promising findings, further studies are needed to evaluate the efficacy of the Hw complex in models of allergen-induced airway inflammation. Such investigations will be necessary to determine therapeutic value under conditions mimicking chronic respiratory diseases.

## 3. Materials and Methods

### 3.1. Chemicals

The citric acid (CA) p.a., histamine, methacholine, salbutamol, and codeine phosphate were obtained from Sigma-Aldrich Chemicals (Lambda Life, Bratislava, Slovakia) and before the experimental procedures were dissolved in 0.9% saline according to the manufacturer’s instructions.

### 3.2. Plant Material and Isolation of (Poly)Phenolic Polysaccharide–Protein Complexes

Ripe dark blue fruits of *Prunus spinosa* L. were harvested in the Kysuce region of Slovakia (Svrčinovec-Bahno district) in November 2015. Botanical identification of blackthorn (*Prunus spinosa* L.) was performed by Dr. Pavol Mereďa from Institute of Botany, Center for Plant Biology and Biodiversity, Slovak Academy of Sciences, Bratislava, Slovakia. Freshly harvested fruits of *Prunus spinosa* L. (~12.5 L) were pitted, blended, and juiced (3.6 kg). The fruit residues were further boiled in 96% ethanol (3 L, 30 min), washed thoroughly with ethanol, and after mixing with distilled water, lyophilized to obtain cell wall material (510 g) for next extraction steps.

The cell wall material (500 g) was stirred three times in distilled water (3 L) containing 0.02% sodium azide at room temperature for 20 h. The extracts were filtered, gradually concentrated, combined, and precipitated into 96% ethanol (1:5 *v*/*v*) containing 1% acetic acid. They were left overnight in a cold room, and precipitates were isolated by centrifugation, washed with ethanol, suspended in distilled water, dialyzed, and freeze-dried to obtain the cold water fraction designated Cw.

The residues after cold water extraction were further extracted three times in hot distilled water (70 °C, 3 L) for 1 h. The extracts were filtered, gradually concentrated, combined, and precipitated in 96% ethanol (1:5 *v*/*v*) containing 1% acetic acid. They were left overnight in a cold room, and precipitates were separated by centrifugation, washed with ethanol, suspended in distilled water, dialyzed, and lyophilized to obtain the hot water fraction designated Hw [5].

### 3.3. General Methods

The contents of carbohydrates, proteins, uronic acids and phenolic substances were determined in the fractions colorimetrically using phenol–sulfuric acid [21], Bradford reagent [22], m-hydroxydiphenyl reagent [23], and Folin–Ciocalteu assays [24].

The quantitative neutral sugar determinations of fractions Cw and Hw were carried out by gas chromatography after their hydrolyses with 2M TFA for 1 h at 120 °C in the form of their alditol acetates [25]. Samples were analysed on a TRACE Ultra Gas Chromatograph (Thermo Scientific, Waltham, MA, USA) equipped with a TG-SQC (Thermo Scientific, Waltham, MA, USA) capillary column (30 m × 0.25 mm × 0.2 µm) at a temperature program of 80 °C (4 min)—(8 °C/min)—160 °C (4 min)—(4 °C/min)—250 °C (20 min), and the flow rate of helium was 0.4 mL/min. The gas chromatograph was coupled with an ITQ 900 mass spectrometer (Thermo Scientific, Waltham, MA, USA) with EI ionization under standard 70 eV electron energy, an emission current of 25 μA, and an ion source temperature of 200 °C. Alditol acetates of monosaccharides Rha, Fuc, Rib, Ara, Xyl, Man, Gal, and Glc were used as standards.

Molecular weight distribution patterns of samples were obtained by SEC-HPLC using an Agilent Technologies 1260 Infinity (Agilent Technologies Inc., Santa Clara, CA, USA), RI and DAD detectors; a tandem of columns HEMA-BIO 1000 and HEMA-BIO 300 (8 mm × 250 mm); a mobile phase of 0.1M NaNO_3_ with a flow rate of 0.4 mL/min; and an injection volume of 25 μL). A set of dextran standards (Mw 5220; 25,500; 72,700; 158,100; 344,800 and 759,400 g/mol, American Polymer Standards Corporation (APSC, Mentor, OH, USA) were used for columns’ calibration. The average molecular weight (Mw) was calculated using Agilent Cirrus GPC software (version 1.2) (Agilent Technologies, Inc., Santa Clara, CA, USA).

NMR spectra of fractions were measured in D_2_O, at 60 °C on a Bruker AVANCE III HD 400 MHz spectrometer equipped with a broad-band BB-(H-F)-D-05-Z liquid N_2_ Prodigy probe (Bruker BioSpin, Germany). For ^1^H and ^13^C NMR spectra, chemical shifts were referenced to TSP-d_4_ (δ 0/0 ppm). For the assignment of signals, advanced techniques of 1D and 2D homo- and hetero-correlated spectroscopy from the Bruker pulse sequence library were applied.

### 3.4. Experimental Animals

This study utilized male Dunkin–Hartley guinea pigs (n = 56) with a body weight ranging from 250 to 300 g. The animals were sourced from a certified breeding facility, Velaz Ltd., in Prague, Czech Republic (CZ 21760118). They were housed in an accredited animal facility under controlled conditions (air-conditioned rooms maintained at 23 ± 2 °C, humidity levels between 50 and 60%, a 12 h light/dark cycle, and continuous access to water and standard laboratory chow), in compliance with Directive 2010/63/EU on the protection of animals used for scientific purposes [26]. Prior to the experiment, the guinea pigs underwent an acclimatization period to minimize stress responses. Animals were kept in groups and provided with paper bedding and shelters as environmental enrichment to support natural behaviour and reduce stress.

Specific inclusion criteria included good general health status and a clearly elicitable cough reflex prior to the experiment. Animals showing signs of ongoing illness or lacking a reliable cough reflex were excluded. These criteria were established *a priori.* The animals were randomly assigned to three control groups (two positive and one negative) and four experimental groups, with each group consisting of eight animals. The treatment groups were as follows.

- Negative control group OVA-, which were administered saline orally at a dose of 1 mL/kg body weight (bw).
- Positive control group COD, which received codeine phosphate orally at a dose of 10 mg/kg bw.
- Positive control group SAL, which were treated with inhaled salbutamol (5 min treatment with 4 µM solution).
- Experimental group Cw 50, which were administered Cw glycoconjugate at a dose of 50 mg/kg bw.
- Experimental group Hw 50, which were treated with Hw sample at a dose of 50 mg/kg bw.
- Experimental group Hw 75, which received Hw glycoconjugate at a dose of 75 mg/kg bw.
- Experimental group Hw 100, which were treated with Hw at a dose of 100 mg/kg bw.

All experimental procedures were approved by the local Ethics Committee (IRB00005636, Decision No. EK 40/2018) and conducted in compliance with Directive 2010/63/EU of the European Parliament and the Council and Slovak regulations on the ethical use of animals in research [26].

### 3.5. The Methodology for Evaluating the Effects of Cw and Hw on Airway Defence Reflexes

#### 3.5.1. Assessment of Antitussive Effect

The methodology used to evaluate the cough reflex, experimentally induced by a chemical tussigen (0.3 M citric acid), has been extensively described in previous studies [27,28]. This method has also been adapted to assess the antitussive effects of plant-derived substances [29,30].

In brief, awake guinea pigs were placed in a double-chamber body plethysmograph designed for small laboratory animals (HSE type 855, Hugo Sachs Elektronik, March, Germany). The plethysmograph consists of separate nasal and thoracic chambers, divided by an elastic ring positioned around the animal’s neck. Changes in pressure recorded within each chamber, corresponding to respiratory and cough-related movements, were displayed as a flow curve.

A citric acid (CA) aerosol was generated using a PARI jet nebulizer (Paul Ritzau, Pari-Werk GmbH, Starnberg, Germany; output 5 L/s, mass median particle diameter 1.2 μm) and applied to the nasal chamber for three minutes. CA induces coughing by stimulating rapidly adapting receptors (RARs), TRPV-1, acid-sensing ion channels, and/or extrapulmonary cough receptors [31]. The number of coughs was recorded during inhalation.

Coughing events were identified based on characteristic changes in the flow curve, visible coughing movements, and the corresponding sound. Two independent trained observers monitored and confirmed each cough event. The number of coughs was assessed before administration of the tested substances and then re-evaluated at 120 and 240 minutes post-administration. The antitussive effects of Cw and Hw were compared to the standard antitussive drug, codeine.

#### 3.5.2. Assessment of the Bronchodilatory Effect

The airway reactivity and bronchodilatory effects of the tested substances were evaluated by measuring changes in specific airway resistance (sRaw). These measurements were recorded before and after administration of the test substances at the same time intervals as the cough assessments.

The sRaw values were calculated using the PULMODYN Data Acquisition Software for Respiratory Studies version 2.0 (Harvard Apparatus, Holliston, MA, USA), based on the method described by Pennock et al. [32]. The calculation was derived from recorded airflow changes in both chambers of the body plethysmograph and the time delay between them.

During the experiment, conscious guinea pigs were exposed to aerosolized contractile mediators—histamine or methacholine (both at a concentration of 1 μM/L)—for 30 s. Following this exposure, sRaw values were recorded for one minute. A three-minute interval was maintained between the administration of the broncho-contracting agent and the sRaw measurement, during which fresh air was insufflated into the nasal chamber.

Baseline sRaw values were recorded before the application of any agent. Subsequent measurements, taken at 60 and 240 min post-administration, were used to assess the effects of Cw, Hw, and salbutamol on airway reactivity.

### 3.6. Statistical Analysis

All data are presented as mean ± standard deviation (SD). Statistical comparisons between experimental groups were performed using a one-way analysis of variance (ANOVA) followed by the Bonferroni’s post hoc test for multiple comparisons. A significance level of *p* < 0.05 was considered statistically significant. Normality of data distribution was assessed by descriptive statistics and graphical methods. As the data met the assumptions for parametric testing, no data transformations were required. Statistical analysis was conducted using GraphPad Prism version 8.0 (GraphPad Software, San Diego, CA, USA).

## 4. Conclusions

Underutilized wild blackthorn (*Prunus spinose* L.) berries, which are a significant source of fibers and phenolic compounds, could represent an alternative source of functional foods with a beneficial effect on human health. (Poly)phenolic polysaccharide–protein complexes were isolated from blackthorn berries by cold and hot water extraction. The complexes differed in their carbohydrate, phenolic, protein and uronic acid contents, while their molecular weights were similar. Both complexes were rich in acidic and highly esterified homogalacturonan, which is usually associated with arabinan or arabinogalactan, components of pectin material. Some galacturonic acids were partly acetylated at the O3 position. Studies of *P. spinosa* (poly)phenolic polysaccharide–protein complexes have provided insights into their composition and have also revealed previously unknown pharmacodynamic properties. One of the key findings of this research is the confirmation of previously undocumented pharmacodynamic effects of the hot water complex (Hw), particularly its ability to suppress cough (similar to the conventional centrally acting antitussive codeine) and to induce bronchodilation (similar to salbutamol). The observed reduction in cough and bronchoconstriction in an experimental guinea pig model suggests that this *P. spinosa* complex could be used as an adjunct in the symptomatic treatment of obstructive airway diseases, e.g., bronchial asthma or COPD. Despite the promising findings, further studies are needed to evaluate the efficacy of Hw complexes in models of allergen-induced airway inflammation. Such investigations will be necessary to determine their therapeutic value under conditions mimicking chronic respiratory diseases.

## Figures and Tables

**Table 1:** Compositional analyses of cold (Cw) and hot water (Hw) fractions of wild blackthorn fruits.

Fractions	Cw	Hw
	(wt%)	(wt%)
Yields	1.0	3.8
Carbohydrate	26.4	32.2
Protein	6.8	12.3
Phenolics	6.2	10.7
Uronic acids	33.7	39.3
*M*_w_ (g/mol)	235,200	218,400
*Neutral sugars ^a^*		
Rhamnose	3.7	4.0
Fucose	0.4	0.8
Arabinose	13.0	11.5
Xylose	1.1	1.1
Mannose	1.3	2.2
Galactose	3.6	8.7
Glucose	3.3	3.9

^a^ The proportion of neutral monosaccharides in the samples (Figure S1, Supplementary Data) was calculated based on the carbohydrate content in Cw (26.4 wt%) and Hw (32.2 wt%) [5].

## Data Availability

The data presented in this study are available upon request from the corresponding author.

## Supplementary Materials

The following supporting information can be downloaded at: https://www.mdpi.com/article/10.3390/ijms26135993/s1.

## Abbreviations

ANOVA	One-way analysis of variance
Ara	Arabinose
AG	Arabinogalactan
ASM	Airway smooth muscle
BC	Butamirate citrate
CA	Citric acid
COD	Codeine
COPD	Chronic obstructive pulmonary disease
Cw	Cold water extract
D_2_O	Deuterium oxide
EI	Electron ionization
ERS	European Respiratory Society
Gal	Galactose
GC-MS	Gas chromatography–mass spectrometry
Glc	Glucose
He	Helium
HPLC	High-performance liquid chromatography
HG	Homogalacturonan
Hw	Hot water extract
HSQC	Heteronuclear single quantum coherence spectroscopy
i.p.	Intraperitoneally
Man	Mannose
Mw	Molecular weight
NaBH_4_	Sodium borohydride
NaNO_3_	Sodium nitrate
NMR	Nuclear magnetic resonance
p.o.	Perorally
Rha	Rhamnose
RG-I	Rhamnogalacturonan
RR	Respiratory rate
SAL	Salbutamol
SEC-HPLC	Size exclusion–high-performance liquid chromatography
sRaw	Specific airway resistance
