# Supplementary material for: Pharmacological Effect of Water-Extractable (Poly)Phenolic Polysaccharide–Protein Complexes from Prunus spinosa L. Wild Fruits
Source: Int J Mol Sci. 2025 Jun 22;26(13):5993. doi: 10.3390/ijms26135993 (PMC12249505; doi:10.3390/ijms26135993)
Supplement: Supplementary file 1 [file ijms-26-05993-s001.zip › ijms-3680216-supplementary.pdf]

Supplementary data

Pharmacological effect of water-extractable (poly)phenolic polysaccharide-protein complexes from *Prunus spinosa* L. wild fruits

Martina Šutovská[1], Miroslava Molitorisová[1], Jozef Mažerik[1], Iveta Uhliariková[2], Peter Capek[2*]

[1] *Department of Pharmacology, Jessenius School of Medicine in Martin, Comenius University in Bratislava, Malá Hora 11161/4B, SK-03601, Martin, Slovakia*

[2] *Institute of Chemistry, Slovak Academy of Sciences, Dúbravská cesta 9, SK-845 38 Bratislava, Slovakia*

** Correspondence: chemcape@savba.sk; Tel.: 00421 59410 220*

Cw fraction

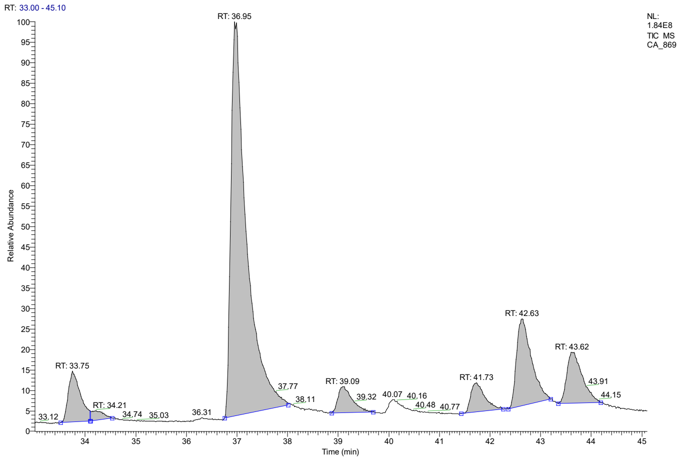

**Cw**	PEAK LIST				
	CA_869.raw				
	RT: 33.00 - 45.10				
	Number of detected peaks: 7				
	Apex RT	Start RT	End RT	Area	Area %
**Per-O-Acetyl-Ramnitol**	33.75	33.51	34.09	383673354.4	6.33
**Per-O-Acetyl-Fucitol**	34.21	34.11	34.53	70933507.06	1.17
**Per-O-Acetyl-Ribitol**	-				
**Per-O-Acetyl-Arabitol**	36.95	36.75	38.01	3816671351	62.94
**Per-O-Acetyl-Xylitol**	39.09	38.86	39.68	234545277.6	3.87
**Per-O-Acetyl-Manitol**	41.73	41.42	42.26	254399880.4	4.2
**Per-O-Acetyl-Galaktitol**	42.63	42.36	43.19	810580518.9	13.37
**Per-O-Acetyl-Glucitol**	43.62	43.35	44.18	492767095.5	8.13

Hw fraction

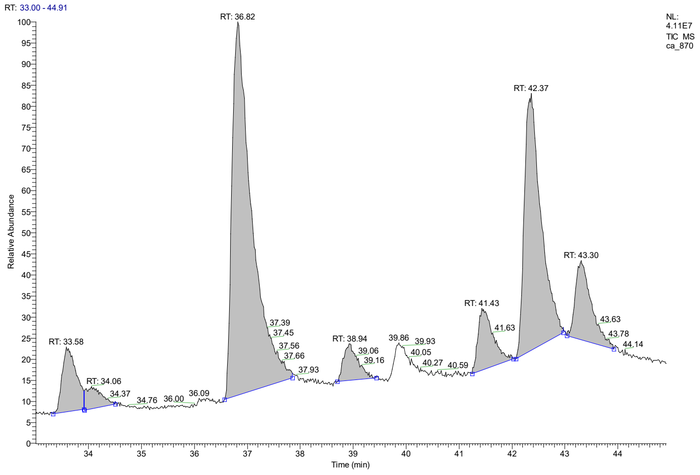

Hw	PEAK LIST				
	ca_870.raw				
	RT: 33.00 - 44.91				
	Number of detected peaks: 7				
	Apex RT	Start RT	End RT	Area	Area %
**Per-O-Acetyl-Ramnitol**	33.58	33.33	33.91	116466361.2	5.8
**Per-O-Acetyl-Fucitol**	34.06	33.92	34.5	43781432.37	2.18
**Per-O-Acetyl-Ribitol**	-				
**Per-O-Acetyl-Arabitol**	36.82	36.57	37.85	947836604.2	47.24
**Per-O-Acetyl-Xylitol**	38.94	38.7	39.43	67720533.47	3.37
**Per-O-Acetyl-Manitol**	41.43	41.24	42.02	118770171.6	5.92
**Per-O-Acetyl-Galaktitol**	42.37	42.08	42.96	548827823.5	27.35
**Per-O-Acetyl-Glucitol**	43.3	43.03	43.92	163154200.9	8.13

Fig. 1S. Sugar analyses (GC) of cold water fraction (Cw) and hot water fraction (Hw) isolated from blackthorn fruits.

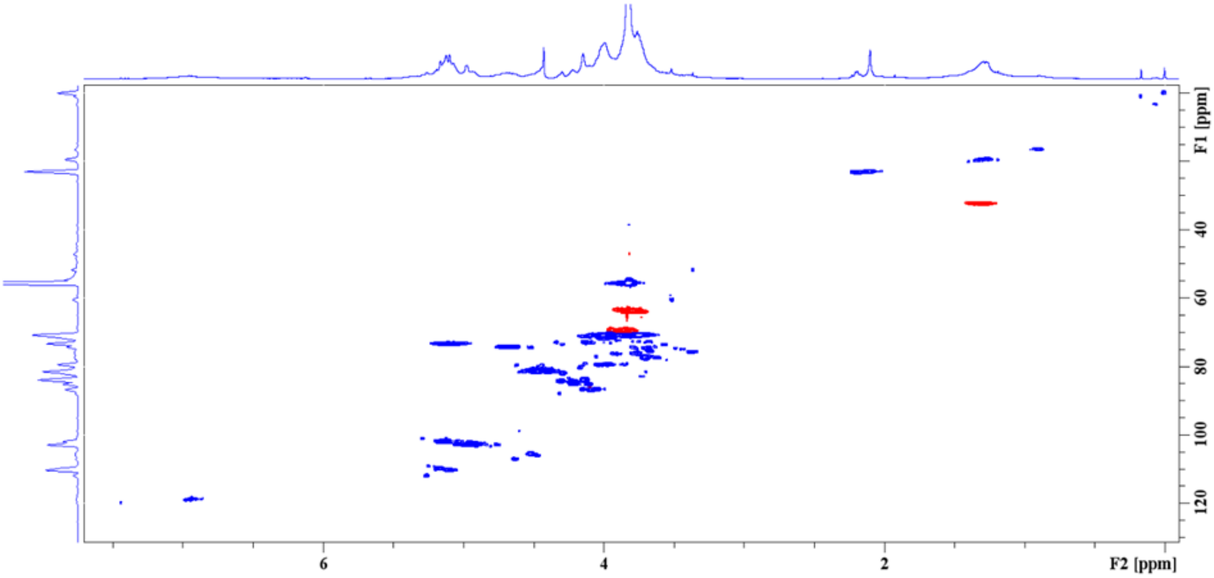

Fig. 2S. Full ^{1}H-^{13}C hetero-correlated HSQC spectrum (CH – blue colour; CH_2 – red colour) of hot water fraction (Hw) of blackthorn fruits.
